# Gremlin1 plays a key role in kidney development and renal fibrosis

**DOI:** 10.1152/ajprenal.00344.2016

**Published:** 2017-01-18

**Authors:** Rachel H. Church, Imran Ali, Mitchel Tate, Deborah Lavin, Arjun Krishnakumar, Helena M. Kok, Jose R. Hombrebueno, Philip D. Dunne, Victoria Bingham, Roel Goldschmeding, Finian Martin, Derek P. Brazil

**Affiliations:** ^1^Centre for Experimental Medicine, Queen’s University Belfast, Belfast, Northern Ireland, United Kingdom;; ^2^Utrecht Medical Centre, Utrecht, The Netherlands;; ^3^Conway Institute, University College Dublin, Dublin, Ireland; and; ^4^Centre for Cancer Research and Cell Biology, Queen's University Belfast, Belfast,Northern Ireland

**Keywords:** Gremlin, kidney, renal fibrosis, development

## Abstract

Gremlin1 (Grem1), an antagonist of bone morphogenetic proteins, plays a key role in embryogenesis. A highly specific temporospatial gradient of Grem1 and bone morphogenetic protein signaling is critical to normal lung, kidney, and limb development. Grem1 levels are increased in renal fibrotic conditions, including acute kidney injury, diabetic nephropathy, chronic allograft nephropathy, and immune glomerulonephritis. We demonstrate that a small number of *grem1^−/−^* whole body knockout mice on a mixed genetic background (8%) are viable, with a single, enlarged left kidney and grossly normal histology. The *grem1^−/−^* mice displayed mild renal dysfunction at 4 wk, which recovered by 16 wk. Tubular epithelial cell-specific targeted deletion of Grem1 (*TEC-grem1-cKO*) mice displayed a milder response in the acute injury and recovery phases of the folic acid model. Increases in indexes of kidney damage were smaller in *TEC-grem1-cKO* than wild-type mice. In the recovery phase of the folic acid model, associated with renal fibrosis, *TEC-grem1-cKO* mice displayed reduced histological damage and an attenuated fibrotic gene response compared with wild-type controls. Together, these data demonstrate that Grem1 expression in the tubular epithelial compartment plays a significant role in the fibrotic response to renal injury in vivo.

gremlin1 (Grem1) is a member of the secreted, cysteine knot DAN family of bone morphogenetic protein (BMP) antagonists. Grem1 is involved in regulating a number of cell functions in developing and adult tissues. A highly specific temporospatial gradient of Grem1 and BMP signaling, which is essential for normal limb, kidney, and lung development, occurs during embryogenesis (1, 13, 19, 20).

Grem1 homozygous knockout (KO) mice on a C57BL/6 background are neonatal lethal due to development abnormalities, including bilateral agenesis of the kidneys, lung defects, and limb malformations (1, 13, 20). Grem1 inhibition of BMP-4 in the metanephric mesenchyme is required to facilitate ureteric bud outgrowth and epithelial branching morphogenesis (19). The lack of kidney development in *grem1^−/−^* mice can be rescued by deletion of one allele of BMP-4, suggesting that an inappropriate “volume” of BMP signaling in the absence of Grem1 leads to renal agenesis (19). Recently, it has been shown that *grem1^−/−^* mice can survive when generated on a mixed genetic background (C57BL/6;FVB). Surviving *grem1^−/−^* mice on this background are smaller, with decreased body weight and a shortened femoral length (1).

Several novel roles for Grem1 have recently been reported, expanding our understanding of the diversity of Grem1 signaling. Grem1 has been suggested to be proangiogenic via activation of vascular endothelial growth factor receptor 2 (VEGFR2) (21). This effect is proposed to occur via Grem1 interactions with heparin sulfate proteoglycans on the surface of endothelial cells (4). Engagement of α_v_β_3_-integrins and formation of α_v_β_3_-integrin-VEGFR2 complexes have also been shown to be involved in Grem1-mediated angiogenesis (27). Grem1 has also been shown to signal in a range of cancer cell lines in a BMP- and VEGFR2-independent manner (14).

Grem1 levels are increased in fibrosis of the kidney, liver, and lung and in colon cancer, pituitary adenoma, and mesothelioma (11, 15, 24, 30, 33). Grem1 levels are increased in diabetic nephropathy (DN), chronic allograft nephropathy, immune glomerulonephritis, and acute kidney injury (AKI) (2, 9, 17, 18, 33). Higher levels of Grem1 expression in the kidney correlate with indexes of renal damage (8, 33). In the context of renal fibrosis, tubular epithelial overexpression of Grem1 in mouse models of AKI and DN resulted in increased renal damage and a more severe phenotype (9, 17). Consistently, Grem1 targeting has shown potential benefits, with both allelic depletion and siRNA targeting of Grem1 protecting against the early sequelae of DN (28, 35). Prophylactic and therapeutic treatment with a Grem1-neutralizing antibody in a murine model of pulmonary arterial hypertension potentiated BMP signaling and reduced pulmonary vascular remodeling and hypertrophy (5).

Since Grem1 expression is increased in renal tubular epithelial cells in damaged human and mouse kidney, we were interested in the specific role of Grem1 in the tubular epithelium in renal injury models. The effect of whole body and tubule epithelial cell-specific deletion of Grem1 in normal and disease states was analyzed. Our results show that Grem1 is required for normal kidney development and that reductions in Grem1, specifically in the tubular epithelium, provide some protection against AKI.

## MATERIALS AND METHODS

All animal experiments were approved in advance by the Queen’s University Animal Welfare and Ethical Review Body and carried out according to the Animal Research Reporting of In Vivo Experiments (ARRIVE) guidelines and the principles of the 3 Rs (replacement, refinement, or reduction of use of animals in research).

### 

#### Generation of whole body Grem1 KO mice.

Whole body *grem1*^+/LacZ^ mice with one allele of *grem1* replaced by a LacZ cassette were provided by Prof. Richard Harland (University of California, Berkeley, CA). The method of Canalis et al. (1) was used to backcross these mice by a single mating to a wild-type FVB strain to generate *grem1*^+/LacZ^ on a mixed C57BL/6J;FVB genetic background. These mice were crossed to yield mice lacking both copies of the *grem1* allele (*grem1^LacZ/LacZ^* or *grem1*^−/−^) at the expected Mendelian frequency of 25%. Mice with two intact alleles of *grem1* (*grem1^+/+^*) were used as wild-type controls.

#### Generation of tubular epithelial cell-specific Grem1 conditional knockout (TEC-grem1-cKO) mice.

The ksp-cadherin-Cre transgenic mice on a C57BL/6J background (stock no. 012237, Jackson Laboratories) express Cre recombinase under the control of the mouse cadherin 16 (Cdh16 or Ksp1.3) promoter (29). The *grem1*^+/fl^ mice on a C57BL/6 background (a generous gift from Prof. Daniel Graf, University of Alberta, Edmonton, AB, Canada) were inbred to generate mice with both copies of the *grem1* allele flanked by *lox*^P^ recombination sequences (*grem1*^fl/fl^). The *grem1*^+/LacZ^ mice were crossed with ksp-cadherin-Cre mice to generate *grem1*^+/LacZ^;ksp-cad-Cre^+/−^ offspring. These mice were then crossed with *grem1*^fl/fl^ mice to generate *TEC-grem1-cKO* tubule-specific deletion (*grem1*^LacZ/Δ^;ksp-cad-Cre^+/−^) mice, where the Δ allele indicates Cre recombinase-mediated deletion of *lox*^P^-flanked *grem1* alleles. These mice were generated at the expected Mendelian frequency of 25%.

#### Genotyping.

Genotyping was performed using genomic DNA isolated from ear biopsies and conventional PCR analysis. Specific oligonucleotides were used to identify the following alleles in whole body *grem1^−/−^* mice: *grem1* [5′-AAAGGTTCCCAAGGAGCCATTCC-3′ (forward) and 5′-AACAGAAGCGGTTGATGATAGTGC-3′ (reverse)] and LacZ [5′-GGTCAATCCGCCGTTTGTTCC-3′ (forward) and 5′-TAGTCACGCAACTCGCCGCACA-3′ reverse)]. The following oligonucleotides were used to identify *TEC-grem1-cKO* mice: *grem1* [5′-AAAGGTTCCCAAGGAGCCATTCC-3′ (forward) and 5′-AACAGAAGCGGTTGATGATAGTGC-3′ (reverse)], LacZ [5′-GGTCAATCCGCCGTTTGTTCC-3′ (forward) and 5′-TAGTCACGCAACTCGCCGCACA-3′ (reverse)], *lox*^P^ [5′-TGGCAGAAAGAATGATACCAGAA-3′ (forward) and 5′-ACAGGTCACACAGTGAATTTGCC-3′ (reverse)], and ksp-cadherin-Cre [5′-GCAGATCTGGCTCTCCAAAG-3′ (forward) and 5′-AGGCAAATTTTGGTGTACGG-3′ (reverse)]. To confirm Cre/*lox*^P^-mediated deletion of *grem1*, genomic DNA was isolated from kidney poles postmortem and analyzed using the following oligonucleotides: 5′-TGGCAGAAAGAATGATACCAG-3′ (forward) and 5′-AACAGGCTAGTATCAGTTACAGC-3′ (reverse). The presence of a 400-bp PCR product confirmed the Cre recombinase-mediated deletion of the *grem1* exon 2 fragment containing the entire *grem1* coding sequence.

#### Immunohistochemistry.

Paraffin-embedded kidney sections (5 μm) from wild-type and *grem1^−/−^* mice were incubated with a Col4a1 antibody (Cell Signaling Technology) according to the manufacturer’s instructions. Col4a1-positive cells were visualized using a FITC-labeled anti-rabbit antibody, and fluorescence intensity was captured using ImageJ software.

#### In situ hybridization.

The RNAscope 2.0 HD detection kit (Advanced Cell Diagnostics, Hayward, CA) was used to perform chromogenic in situ hybridization of Grem1 on formalin-fixed paraffin-embedded mouse colon sections. Briefly, paraffin-embedded colon sections were cut at 4 μm, air-dried overnight, baked at 60°C for 1 h, dewaxed, and air-dried before pretreatments. For all probes, a standard pretreatment protocol was employed according to the manufacturer’s instructions. The specifications of the Grem1 RNAscope probe employed in this study (Mm-Grem1, catalog no. 314741) were as follows: probe region begin, 398; probe region end, 1359. Specific probe binding sites were detected by the RNAscope 2.0 HD detection kit based on 3,3′-diaminobenzidine substrate, with intensity of brown staining as a surrogate measurement of mRNA abundance. Semiquantitative pathology-based image analysis was carried out on tissue sections from three wild-type and three *grem1^−/−^* mice, with positive or negative scoring recorded by three independent observers blinded to tissue labels. All slides were then scanned using an Aperio scanner at ×40 resolution and uploaded to the Queen’s University Belfast digital pathology suite (PathXL), where images were captured.

#### Induction of AKI.

Female wild-type and *TEC-grem1-cKO* mice were injected intraperitoneally with a single dose of vehicle (0.3 M sodium bicarbonate) or folic acid (FA, 250 mg/kg; Sigma, Poole, UK) on *day 2* and FA (100 mg/kg in 0.2 M sodium bicarbonate) on *day 14*. Blood was collected during the study by tail vein bleed and at the end of the study by cardiac puncture.

#### Biochemical assays.

Serum creatinine was measured using a creatinine colorimetric assay kit (BioVision, Milpitas, CA), and serum urea [blood urea nitrogen (BUN)] was measured using a QuantiChrom Urea Assay kit (BioAssay Systems, Hayward, CA), according to the manufacturers’ protocols.

#### Histology.

Harvested kidneys were fixed in 10% formalin or 4% (wt/vol) paraformaldehyde and incubated for 24 h at room temperature. Tissues were processed and embedded in paraffin wax as previously described (28), sectioned, and placed on Superfrost glass slides (Thermo Fisher Scientific, Poole, UK). Sections were stained using standard histology protocols for hematoxylin and eosin and Masson’s trichrome. Stained sections were visualized using a Nikon Lucia cell imaging system and imaged using NIS-Elements software.

#### Pathology scoring.

Sections were blinded and stained with periodic acid-Schiff-diastase. Slides were rated individually on the basis of tubular damage, inflammation, fibrosis, and casts and scored for the degree of the parameter being measured, combined with the percentage of the parameter covering the whole slide. Percentage of positive pathology was graded on a scale of 0–5 as follows: 0 (0%), 1 (0–10%), 2 (10–25%), 3 (25–50%), 4 (50–75%), 5 (75–100%).

Tubular damage was scored on the basis of tubules that were dilated in both length and width. Inflammation was determined by the presence of cells in the interstitial area between tubules. Tubular casts were detected in dilated tubules by the presence of pink staining, indicating a large amount of protein. Interstitial fibrosis was determined by a meshed matrix between tubules that stained pink.

#### Real-time PCR.

RNA was extracted from kidney tissue using the TRIreagent (Sigma-Aldrich) method. Total RNA (1 μg) was reverse-transcribed, and TaqMan PCR was performed using specific TaqMan probes (Roche Applied Science) for Grem1 (assay ID 314702), collagenase type 4 α_1_-subunit (Col4a1; assay ID 300652), vimentin (assay ID 310719), fibronectin (assay ID 300578), and plasminogen activator 1 (PAI-1; assay ID 310686). Analysis was carried out using the comparative threshold (ΔΔC_t_) method and normalized to an average of 18S (assay ID 307906) and β-actin (assay ID 300236) levels. Real-time PCR was carried out on a light cycler (model 480, Roche).

#### Statistical analysis.

Statistical analysis was carried out using GraphPad Prism software, and graphs were generated using Prism 5.0 (GraphPad, San Diego, CA). Student’s unpaired *t*-test was performed to determine significant differences between two normally distributed groups. Student’s paired *t*-test was performed to determine significant difference when a parameter relating to the same group was compared after a specific treatment. A one-way analysis of variance (ANOVA) with a Bonferroni’s post hoc test was performed to compare any differences between more than two group means. *P* < 0.05 was considered to be statistically significant.

## RESULTS

### 

#### Whole body grem1^−/−^ mice develop a single, enlarged kidney.

**Fig. 1.**
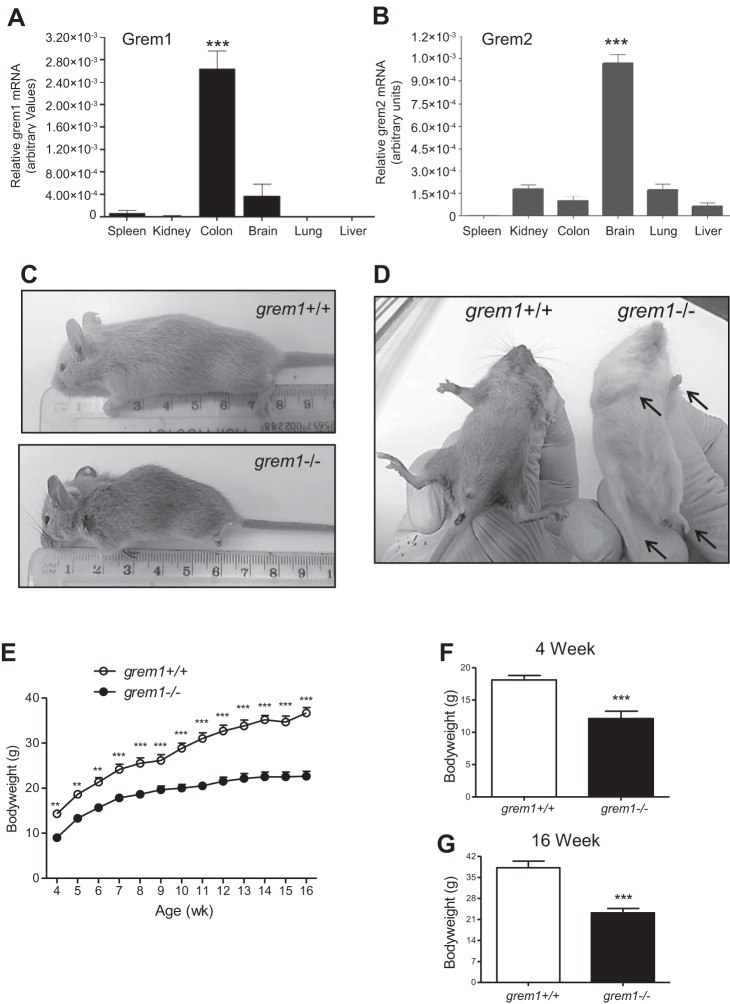
Gremlin1 (Grem1)-deficient (*grem1^−/−^*) mice are smaller and have deformed limbs. *A* and *B*: mouse tissues were analyzed using PCR-RT and specific TaqMan probes for Grem1 or Grem2; 18S and β-actin were used as housekeeping controls. Values (means ± SE, *n* = 8) are shown as relative gene expression [2^−ΔΔCt^ (gene − housekeeping gene average)]. *C*: *grem1^−/−^* mice are smaller than wild-type (*grem^+/+^*) littermates. *D*: *grem1^−/−^* mice display limb deformities, as indicated and previously described (13). *E*: growth curves for 4- to 16-wk-old wild-type and *grem1^−/−^* mice. *F* and *G*: *grem1^−/−^* mice have a significantly lower body weight than wild-type littermates at 4 wk (*n* = 8) and 16 wk (*n* = 5). Values are means ± SE. **P < 0.01, ***P < 0.001 (by Student’s unpaired *t*-test).

**Fig. 2.**
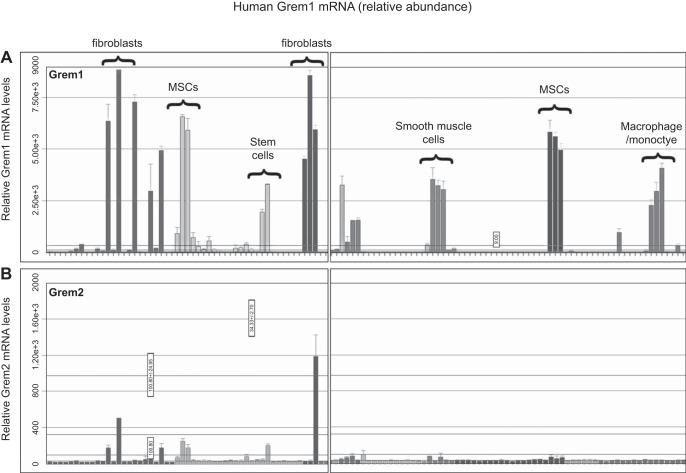
Expression of Grem1 in human tissues and primary cells. Relative levels of Grem1 and Grem2 mRNA were identified on the BioGPS platform (www.bioGPS.org). *A* and *B*: levels of expression in a range of human tissues were measured via gene array and plotted. Tissues and cells with high levels of Grem1 expression are indicated. MSCs, mesenchymal stem cells.

Grem1 and its homolog Grem2 [protein related to Dan and Cerberus (PRDC)] display distinct patterns of expression in mouse tissue. Using quantitative PCR, we identified the highest levels of Grem1 mRNA in colon and lower levels in brain (Fig. 1*A*). In contrast, Grem2 is highest in brain and lower in kidney and lung (Fig. 1*B*). Online analysis of Grem1 expression in human tissues using the BioGPS platform (www.bioGPS.org) confirmed that levels of Grem1 are highest in smooth muscle, small intestine, colon, and uterus (Fig. 2*A*). In addition, high levels of Grem1 were detected in human fibroblasts, astrocytes, endothelial cells, mesenchymal stem cells (MSCs), and monocytes (Fig. 2*A*, and data not shown). Grem2 mRNA distribution was more limited, with high levels detected in fibroblasts, liver, and breast stroma (Fig. 2*B*, and data not shown). Both Grem1 and Grem2 are widely expressed in a range of brain regions (data not shown).

**Fig. 3.**
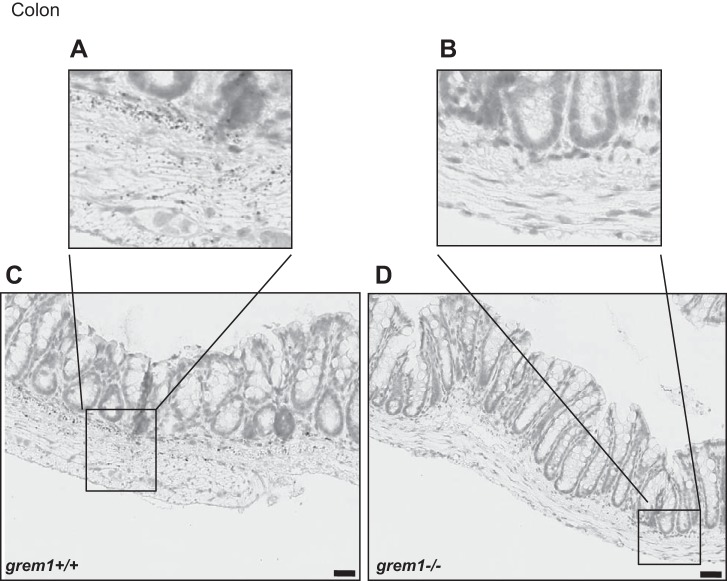
Grem1 mRNA is detected in mouse colon. RNA in situ hybridization-based gene expression of Grem1 was detected using RNAscope probes and 3,3′-diaminobenzidine staining to visualize Grem1-positive cells in *grem1^+/+^* (*A* and *C*) and *grem1^−/−^* (*B* and *D*) mice. Grem1 staining was confined to the muscularis mucosae and submucosal regions of the mouse colon in the wild-type mouse. In contrast, no signal was detected in colon sections from *grem1^−/−^* mice, as assessed by blinded assessment of sections by 3 independent observers. Images, which represent findings from 4 wild-type and 4 *grem1^−/−^* mice, were captured using Aperio slide-scanning technology at ×19 (*A* and *B*) and ×27 (*C* and *D*) magnification. Scale bars = 30 µm.

Whole body deletion of *grem1^−/−^* in mice on a C57BL/6 background leads to death at postnatal *day 2* (*P2*) due to the absence of kidneys (19, 20). Canalis and colleagues reported that a single backcross onto an FVB background allowed some *grem1^−/−^* mice to survive into adulthood (1). We employed this strategy and generated *grem1*^+/−^ mice on a mixed C57BL/6J;FVB background. Crossing these mice led to the expected Mendelian frequency of *grem1*^+/+^, *grem1*^+/−^, and *grem1^−/−^* offspring (Table 1). As expected, the majority of the *grem1*^−/−^ mice died at *P2*; only 8% survived to adulthood (Table 1). Surviving *grem1*^−/−^ mice were smaller and displayed the previously described defects in fore- and hindlimb formation (Fig. 1, *C* and *D*). Weight at birth was lower in *grem1^−/−^* than wild-type mice, and *grem1^−/−^* mice maintained a growth rate similar to that of wild type mice up to 7–8 wk, when their body weight plateaued (Fig. 1*E*). At 16 wk, wild-type mice weighed 36.7 ± 4.9 g and *grem1^−/−^* mice weighed 22.7 ± 3.2 g (*P* < 0.001; Fig. 1*G*). Consistent with high levels of Grem1 detected in the colon (Fig. 1*A*), RNA in situ hybridization detected Grem1 in the muscularis mucosae and submucosal regions of wild-type mouse colon, but not in *grem1^−/−^* sections (Fig. 3) (7). Gross colon histology was normal, and *grem1^−/−^* mice had elevated levels of pSmad1/5 in colon sections, consistent with amplified BMP signaling resulting from Grem1 deletion (data not shown). These data suggest that Grem1 expression is maintained in the colon in the adult mouse, where it antagonizes BMP signaling.

**Table 1.** Outcome of grem1^+/−^ × grem1^+/−^ crosses

Mating	Total No. of Mice	*grem1*^+/+^ Mice	*grem1*^+/−^ Mice	*grem1*^−/−^ Mice	*grem1*^−/−^ Mice That Died at *P2*	*grem1*^−/−^ Mice That Survived
*grem1*^+/−^ × *grem1*^+/−^	202	51 (25.24%)	100 (49.50%)	50 (24.75%)	46 (92%)	4 (8%)

Values represent number of offspring, with percentage of total in parentheses. *P2*, postnatal *day 2*.

**Fig. 4.**
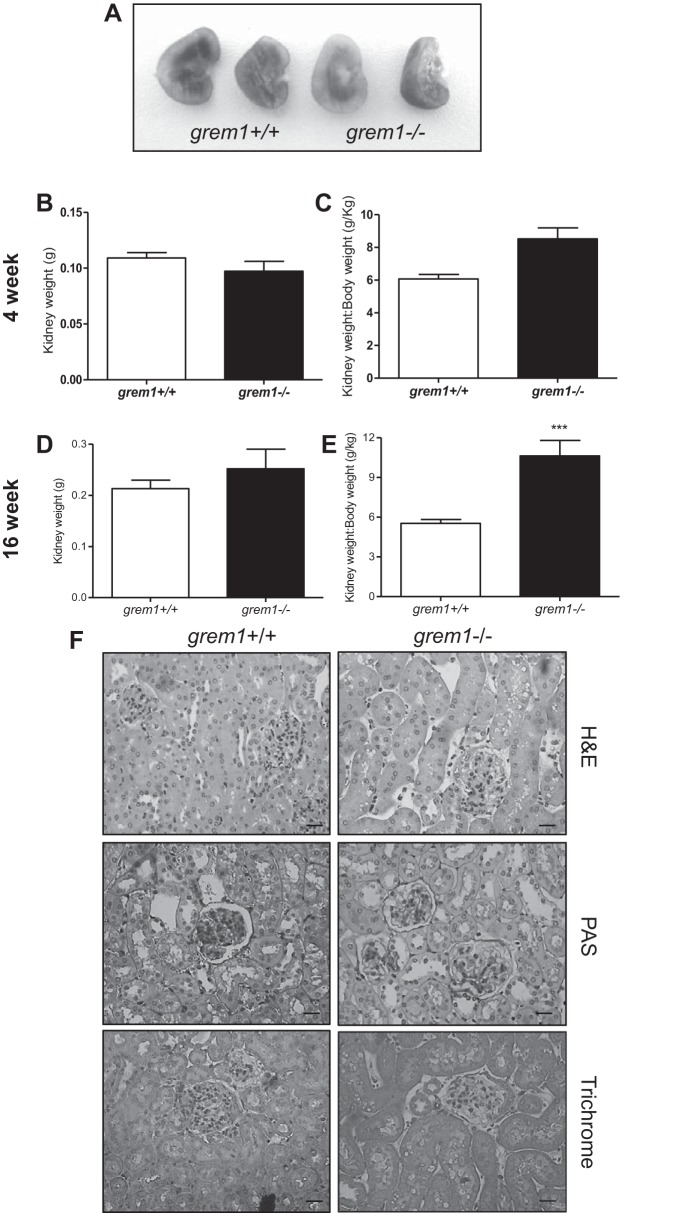
Changes in kidney size, but not structure, in *grem1^−/−^* mice. *A*: representative images of kidneys of *grem1^+/+^* and *grem1^−/−^* mice at 16 wk. *B–E*: kidney weights were recorded and plotted for *grem1^+/+^* and *grem1^−/−^* mice at 4 and 16 wk or normalized to body weight. Values are means ± SE (*n* = 5–8). ***P < 0.001 (by Student’s unpaired *t*-test). *F*: paraffin-fixed kidney sections were cut at 5 µm and stained using hematoxylin and eosin (H&E), periodic acid-Schiff (PAS), or Masson’s trichrome protocol. Scale bars = 70 µm. Stained slides were imaged on a Lucia light microscope. Representative images from *grem1^+/+^* and *grem1^−/−^* mice are shown at ×20 magnification.

Postmortem analysis revealed that *grem1^−/−^* mice that died at *P2* were anephric, with no evidence of kidney development (data not shown). In contrast, the majority of surviving *grem1^−/−^* mice developed a single, enlarged left kidney (Table 2, Fig. 4, *A–E*). Although the body weight of the *grem1^−/−^* mice was only ∼60% of that of wild-type mice at 16 wk, the left kidney of *grem1^−/−^* mice was slightly larger: 213 ± 55 mg (wild-type) vs. 226 ± 87 mg (*grem1*^−/−^) (Fig. 4*D*). Consistently, kidney weight-to-body weight ratios for *grem1^−/−^* mice were also elevated at 4 wk, with a more pronounced difference at 16 wk in *grem1^−/−^* than wild-type mice: 5.534 ± 0.973 g/kg (wild-type) vs. 9.611 ± 3.04 g/kg (*grem1*^−/−^) (*P* < 0.001; Fig. 4*E*). These data indicate that a minority of *grem1^−/−^* mice survive and are smaller, with an enlarged left kidney.

**Table 2.** Kidney distribution in survivor grem1^−/−^ mice

Mouse No.	Age of Cull, wk	Left Kidney	Right Kidney
*1*	4	Yes	No
*2*	4	Yes	No
*3*	4	Yes	No
*4*	4	Yes	No
*5*	4	Yes	No
*6*	4	Yes	No
*7*	4	Yes	No
*8*	4	Yes	No
*9*	16	Yes	No
*10*	16	No	Yes
*11*	16	Yes	No
*12*	16	Yes	Yes
*13*	16	Yes	No

**Fig. 5.**
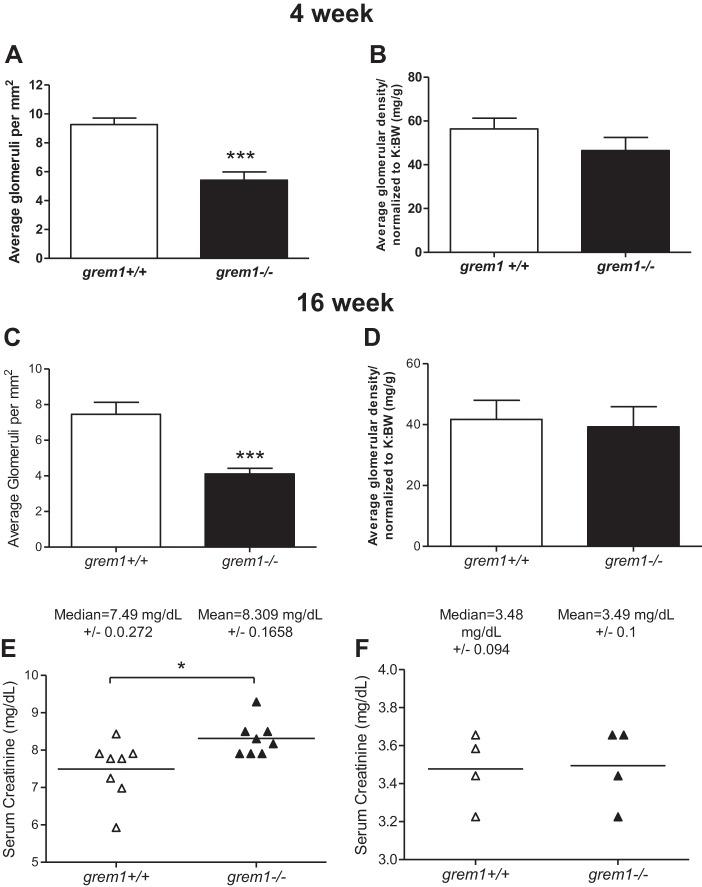
Reduced glomerular density, but similar glomerular number, in *grem1^−/−^* kidney. *A–D*: glomeruli in *grem1^+/+^* and *grem1^−/−^* mouse kidneys were counted. Glomerular number per mm^2^ (density) and total glomeruli normalized to kidney weight-to-body weight ratio (K:BW) were calculated at 4 and 16 wk. Values are means ± SE (*n* = 5–8). ***P < 0.001 (by Student’s unpaired *t*-test). *E* and *F*: serum from *grem1^+/+^* and *grem1^−/−^* mice was isolated and analyzed for serum creatinine at 4 wk (*E*) and 16 wk (*F*). Values are medians ± SE (*n* = 4–8). *P < 0.05 (by Student’s unpaired *t*-test).

**Fig. 6.**
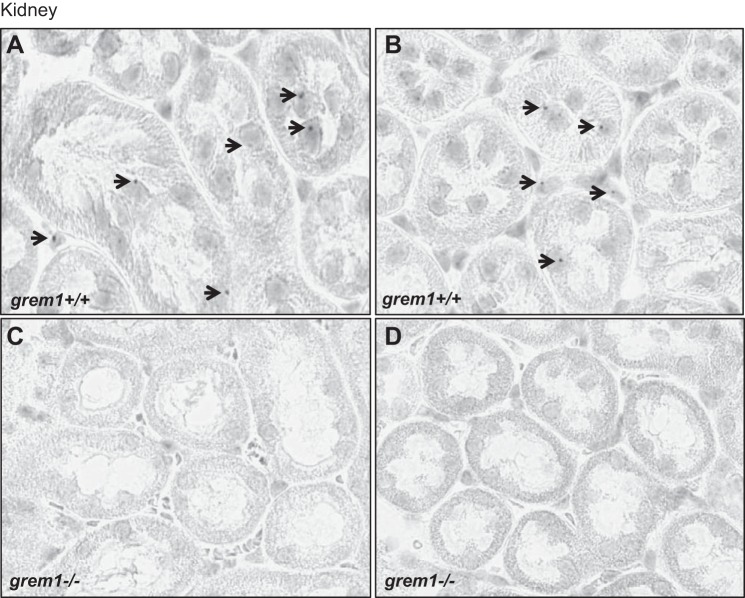
Grem1 mRNA expression in mouse kidney. *A–D*: RNA in situ hybridization-based gene expression of Grem1 was detected using RNAscope probes and 3,3′-diaminobenzidine staining to visualize Grem1-positive cells in *grem1^+/+^* and *grem1^−/−^* mice. Negligible signal was detected in *grem1^+/+^* mice, and no staining was observed in *grem1^−/−^* mice, as assessed by blinded assessment of sections by 3 independent observers. Images, which represent findings from 4 wild-type and 4 *grem1^−/−^* mice, were captured using Aperio slide-scanning technology at ×27 magnification. Scale bars = 30 µm.

The single left kidney from *grem1^−/−^* mice displayed grossly normal histology, with no evidence of glomerular or tubular defects or increased glycoprotein or collagen secretion (Fig. 4*F*). Glomerular density in the single *grem1^−/−^* kidney was reduced compared with the wild-type kidney at 4 and 16 wk: 9.26 (wild-type) vs. 5.41 (*grem1^−/−^*) glomerulae/mm^2^ at 4 wk and 7.46 (wild-type) vs. 4.11 (*grem1^−/−^*) glomerulae/mm^2^ at 16 wk (Fig. 5, *A* and *C*). However, the overall number of glomerulae was comparable between the two genotypes when the glomerular density was normalized to kidney weight-to-body weight ratio, suggesting that the increased size of the single *grem1^−/−^* kidney may be due to expansion of the interstitial compartment. Serum creatinine levels were elevated in *grem1^−/−^* vs. wild-type mice at 4 wk, suggesting a mild renal dysfunction at this time point (Fig. 5*E*). However, by 16 wk, serum creatinine levels had returned to normal (Fig. 5*F*), suggesting a delay in the capacity of the single *grem1^−/−^* kidney to cope with the filtration demands of the developing mouse. Furthermore, RNA in situ hybridization of *grem1^+/+^* and *grem1^−/−^* kidneys revealed some low-level, particulate staining (Fig. 6, *A* and *B*). Conversely, no staining was observed in *grem1^−/−^* kidney (Fig. 6, *C* and *D*), as expected, enabling it to be used as a negative control.

#### Grem1^−/−^ mice have normal retinal vascular development and visual responses.

**Fig. 7.**
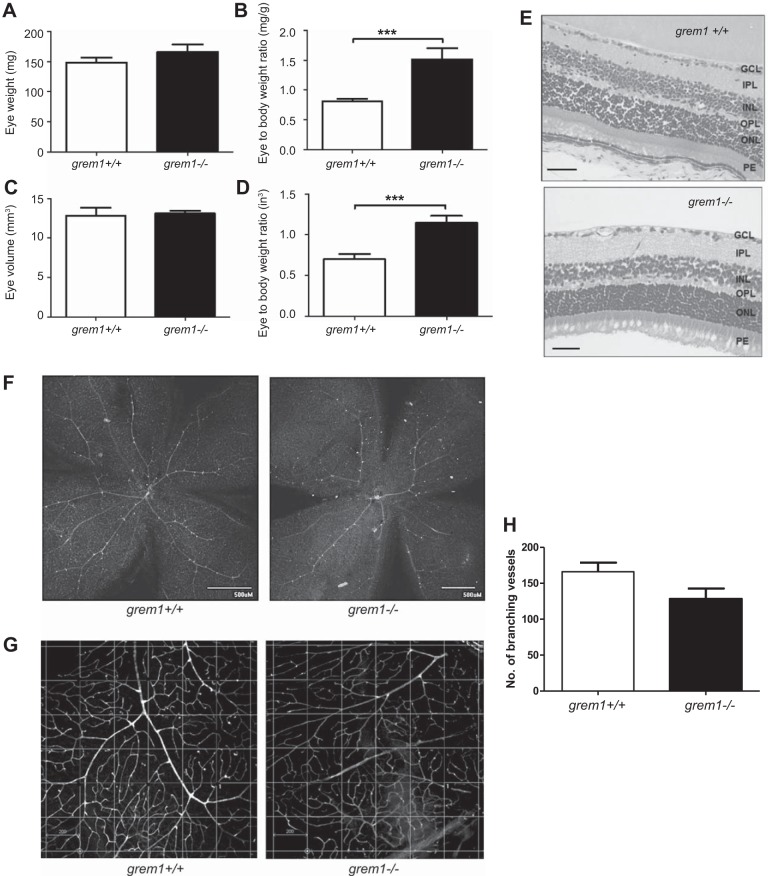
In *grem1^−/−^* mice, eye weight-to-body weight ratios are increased and retinal vascular branching is normal. *A–D*: eye weight and eye volume of wild-type and *grem1^−/−^* mice were measured at harvest (*A* and *C*) or normalized to body weight (*B* and *D*). Values are means ± SE (*n* = 8). ***P < 0.001 (by Student’s unpaired *t*-test). *E*: H&E staining of eye sections from *grem1*^+/+^ and *grem1^−/−^* mice at ×40 magnification. Scale bars = 200 µm. GCL, ganglion cell layer; IPL, inner plexiform layer; INL, inner nuclear layer; OPL, outer plexiform layer; ONL, outer nuclear layer; PE, posterior end. *F*: retinal flat mounts from *grem1*^+/+^ and *grem1^−/−^* mice were stained with isolectin B, and vessels were visualized using FITC-labeled anti-rabbit antibody. Images are shown at ×4 magnification. *G*: defined area within each quadrant of the flat-mount image was selected, and numbers of branching vessels were counted for *grem1*^+/+^ and *grem1^−/−^* mice. Images were obtained at ×10 magnification. Scale bars = 200 µm. *H*: average number of branching vessels within the defined quadrants for wild-type and *grem1^−/−^* retinas. Values are means ± SE (5 images per retina, 5 mice per group). *P* = 0.055 (by Student’s unpaired *t*-test).

A novel role for Grem1 as a proangiogenic activator of VEGFR2 has been proposed in recent years (4, 21, 27). Based on these data, we hypothesized that *grem1^−/−^* mice may display developmental defects in vascular development. Examination of eyes from wild-type and *grem1*^−/−^ mice demonstrated that, despite a 30–40% reduction in body size of *grem1^−/−^* mice (Fig. 1, *E–G*), eye weight and volume were equivalent in *grem1^−/−^* and wild-type mice (Fig. 6). Gross retinal structure appeared normal, and retinal thickness, as measured from the ganglion cell layer to the posterior end of the outer nuclear layer (∼140 μm), was not significantly different between wild-type and *grem1^−/−^* mice (data not shown). Quantitation of the number of branching vessels within the retina showed no significant difference in wild-type vs. *grem1^−/−^* mice (Fig. 7, *F–H*). Consistently, there were no significant differences in visual function of wild-type and *grem1^−/−^* mice, as assessed by electroretinogram (data not shown).

#### TEC-grem1-cKO mice display milder fibrosis responses to FA injury.

**Fig. 8.**
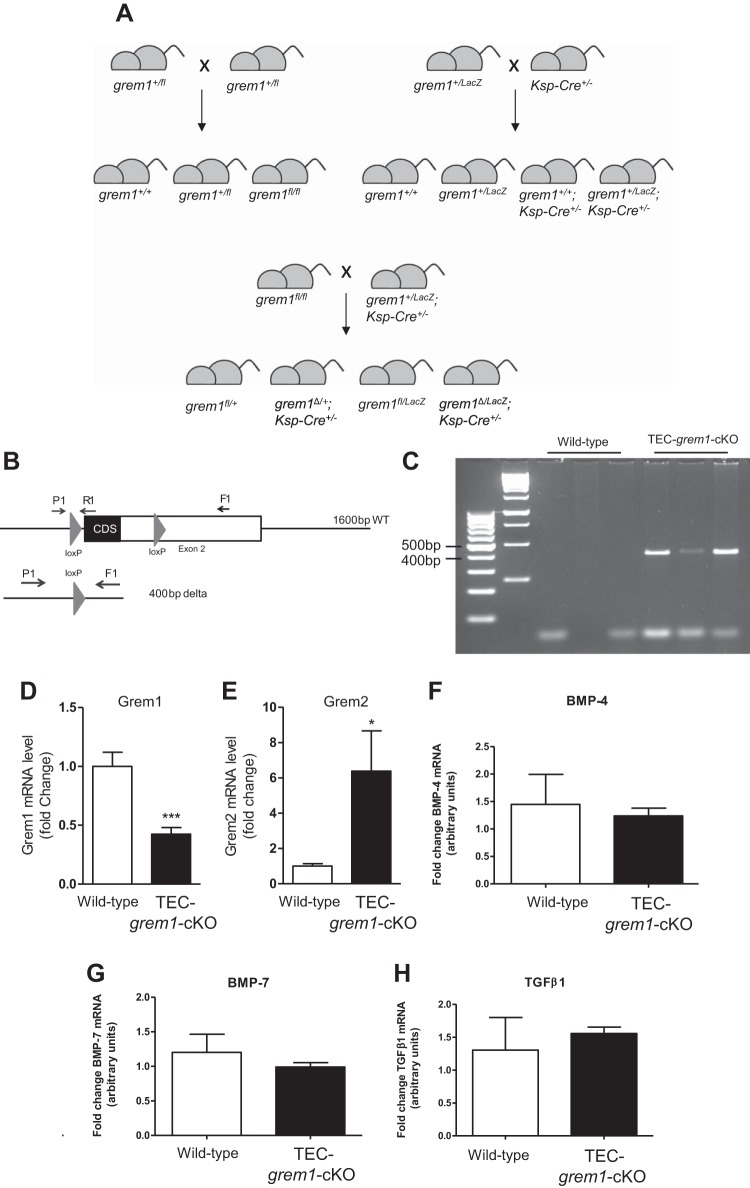
Generation and identification of *TEC-grem1-cKO* mice. *A*: F1 generation *grem1*^+/LacZ^;ksp-Cre^+/−^ mice were crossed with *grem1*^fl/fl^ mice to generate tubule-specific Grem1 deletion mice (*grem1*^Δ/LacZ^;ksp-cad-Cre^+/−^ or *TEC-grem1-cKO*). *B* and *C*: genomic DNA was isolated from kidney poles of wild-type (*grem1*^+/fl^) and *TEC-grem1-cKO* mice postmortem and analyzed by conventional PCR. The presence of a 400-bp PCR product confirmed the Cre recombinase-mediated deletion of the *grem1* exon 2 fragment containing the entire *grem1* coding sequence. *D–H*: levels of Grem1, Grem2, bone morphogenetic protein (BMP)-4, BMP-7, and TGFβ1 were measured using RT-PCR with specific TaqMan probe sets. Data were analyzed using the ΔΔC_t_ method and normalized to the mean of 2 housekeeping genes (18S and β-actin). Values (means ± SE, *n* = 10) are plotted as fold change, with Grem1 and Grem2 levels in wild-type set to 1. *P < 0.05, ***P < 0.001 (by Student’s unpaired *t*-test).

We used the ksp-cadherin-Cre mouse generated by the Igarashi laboratory to drive *lox*^P^-mediated deletion of Grem1 in tubular epithelial cells (12). A summary of the mating strategy used to produce *TEC-grem1-cKO* mice is shown in Fig. 8*A*. To confirm Cre-mediated deletion of *grem1*, genomic DNA was isolated from kidney poles postmortem and analyzed by conventional PCR. The presence of a 400-bp PCR product confirmed the Cre recombinase-mediated deletion of the *grem1* exon 2 fragment containing the entire *grem1* coding sequence (Fig. 8, *B* and *C*). Critically, Grem1 mRNA levels were significantly reduced in the kidney of *TEC-grem1-cKO* mice compared with wild-type mice, providing evidence supporting Grem1 TEC deletion (Fig. 8*D*). Grem2 expression was significantly increased in *TEC-grem1-cKO* mice compared with wild-type controls, suggesting compensatory changes in these related BMP antagonists (Fig. 8*E*). Interestingly, no significant difference was observed in BMP-4, BMP-7, or TGFβ1 expression in kidneys of wild-type or *TEC-grem1-cKO* control mice, suggesting that TEC deletion of Grem1 does not alter BMP or TGFβ1 signaling under normal conditions (Fig. 8, *F–H*).

**Fig. 9.**
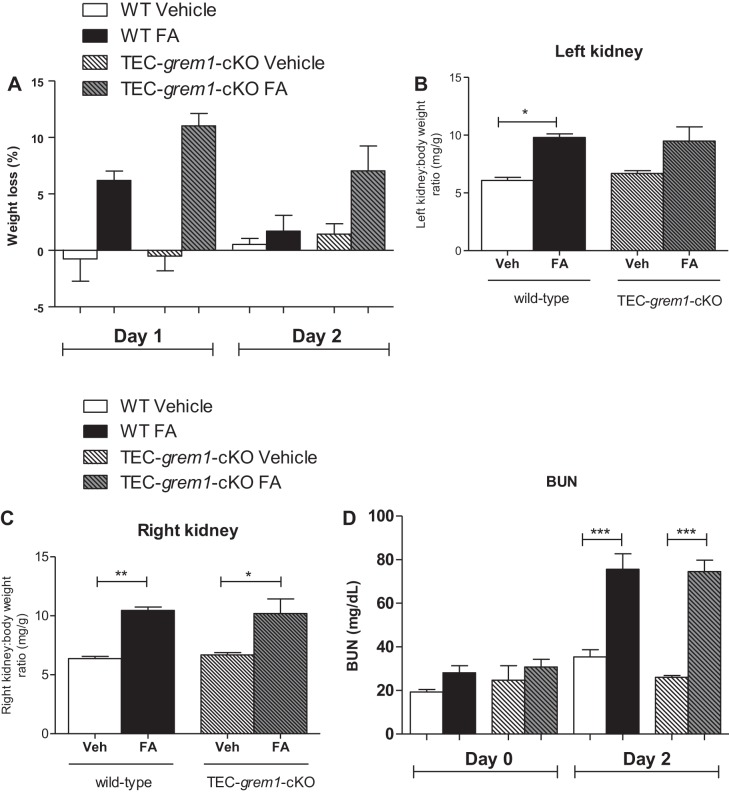

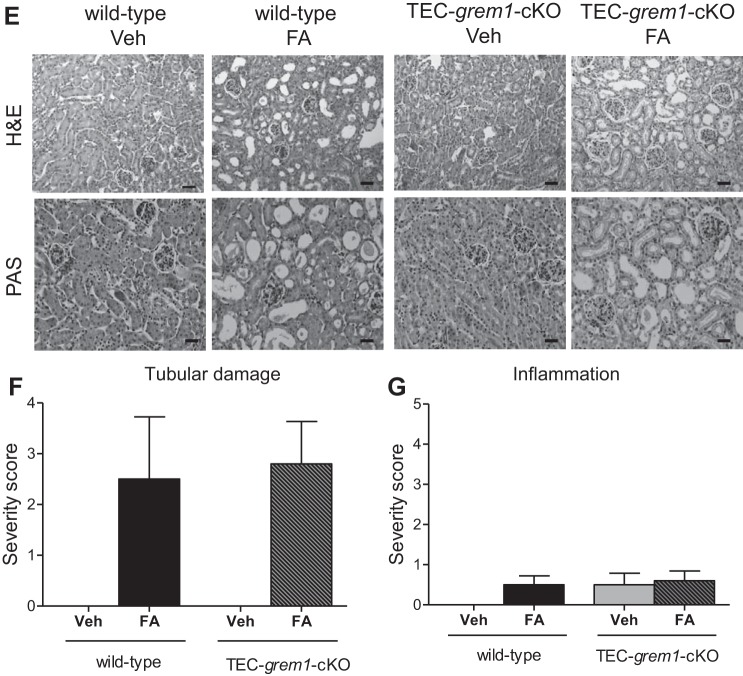
Folic acid (FA) induces acute kidney injury in wild-type and *TEC-grem1-cKO* mice. *A*: mice were weighed and body weights were recorded on *day 0* and at *days 1* and *2* post-vehicle (Veh) or FA (250 mg/kg) injection. Weight loss at *days 1* and *2* was plotted as percentage of *day 0* body weight. Mice were divided into 4 subgroups based on treatment: wild-type (WT) vehicle (*n* = 4), WT FA (*n* = 6), *TEC-grem1-cKO* vehicle (*n* = 4), and *TEC-grem1-cKO* FA (*n* = 6). *B* and *C*: left and right kidney weights were normalized to body weight and plotted for each group as described in *A*. Values are means ± SE. *P < 0.05, **P < 0.01 (by 1-way ANOVA with Bonferroni's multiple comparison test). *D*: serum was isolated at *days 0* and *2* and analyzed for blood urea nitrogen (BUN) for each group. Values are means ± SE. ***P < 0.001, vehicle vs. FA (by Student’s unpaired *t*-test). *E*: formalin-fixed kidney samples were processed and embedded in paraffin wax. Sections were cut at 5 µm and stained using H&E or PAS protocol. Stained slides were imaged on a Lucia light microscope. Representative images from each group are shown at ×20 magnification. Scale bars = 100 µm. *F* and *G*: sections on glass microscope slides were stained with PAS, blinded, and scored on the basis of tubular damage and inflammation as percent positive: 0 (0%), 1 (0–10%), 2 (10–25%), 3 (25–50%), 4 (50–75%), 5 (75–100%). Values are means ± SE.

Mice were harvested 2 days after FA administration to assess the extent of AKI. Wild-type, but not *TEC-grem1-cKO*, mice displayed significant weight loss at *days 1* and *2* post-FA administration (Fig. 9*A*). Most mice in the FA-treated group appeared very unwell at *day 2*, likely due to FA-induced acute renal failure and dehydration. Postmortem, left and right kidney weight-to-body weight ratios were significantly increased in wild-type FA-treated mice (Fig. 9, *B* and *C*). In contrast, this increase was only significant for the right kidney in *TEC-grem1-cKO* mice (Fig. 9*C*). Basal BUN of ~20–30 mg/dl was observed in wild-type and *TEC-grem1-cKO* mice, suggesting that tubular deletion of Grem1 does not alter basal renal filtration (Fig. 9*D*, *day 0*). Importantly, there was no significant difference in serum BUN levels at *day 2* post-FA injection between wild-type and *TEC-grem1-cKO* mice (Fig. 9*D*). A similar trend was observed when serum creatinine was analyzed (data not shown). Histological staining of kidney sections revealed that both wild-type and *TEC-grem1-cKO* mice treated with FA displayed severe kidney injury at *day 2*, with massive tubular dilation and an increased presence of apoptotic cells surrounding necrotic tubules (Fig. 9, *E* and *F*). Minimal tubular casts and fibrosis were observed at *day 2* in both phenotypes (Fig. 9*E*). These data suggest that tubular deletion of Grem1 in mice does not exert a protective effect in the acute phase of FA-induced injury (Fig. 9, *D–F*).

**Fig. 10.**
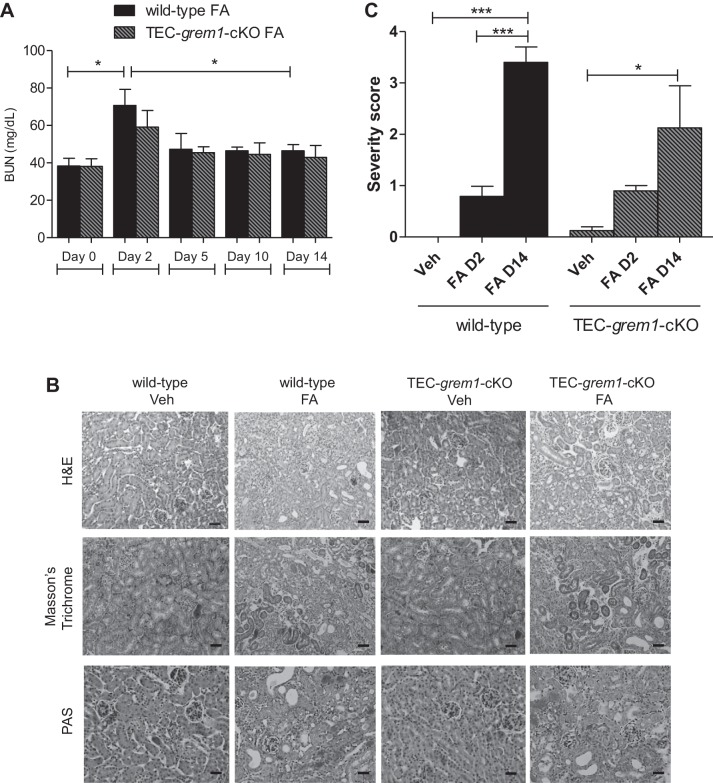
*TEC-grem1-cKO* mice display an attenuated renal fibrosis in FA-induced nephropathy. *A*: blood samples were collected from FA-injected mice at *days 0, 2, 5*, and *10* by tail vein bleed and at *day 14* by cardiac puncture. Serum was isolated and analyzed for BUN. Values are means ± SE [*n* = 5 (wild-type) and 4 (*TEC-grem1-cKO*) mice]. *P < 0.05 between groups at each time point (by Student’s unpaired *t*-test) and for different time points but the same genotype (Student’s paired *t*-test). *B*: formalin-fixed kidney samples were processed and embedded in paraffin wax. Sections were cut at 4 µm and stained using H&E, Masson’s trichrome, or PAS protocol. Scale bars = 100 µm. Stained slides were imaged on a Lucia light microscope. Representative images from each group are shown at ×20 magnification. Sections on microscope glass slides were stained with PAS, blinded, and scored for tubular damage, inflammation, fibrosis, and tubular casts. *C*: combined score of overall damage was plotted for wild-type and *TEC-grem1-cKO* vehicle (Veh), FA *day 2* (FA D2), or FA *day 14* (FA D14). Slides were graded as percent positive: 0 (0%), 1 (0–10%), 2 (10–25%), 3 (25–50%), 4 (50–75%), 5 (75–100%). Values are means ± SE. *P < 0.05, ***P < 0.001 (by 1-way ANOVA with Bonferroni's multiple comparison test).

Because 250 mg/kg FA was associated with a high mortality rate, 100 mg/kg FA was used to study the effects to the *day 14* end point. In response to 100 mg/kg FA, wild-type mice displayed significantly increased serum BUN levels at *day 2* that significantly decreased at *day 14*, indicating renal recovery (Fig. 10*A*). In contrast, *TEC-grem1-cKO* mice exhibited a more modest increase in serum BUN that did not reach statistical significance (Fig. 10*A*). *Day 14* histology revealed FA-induced kidney damage in both groups, with the presence of some dilated tubules and collagen and glycoprotein staining (Fig. 10*B*). Sections from *days 2* and *14* were scored according to severity using the parameters tubular damage, inflammation, fibrosis, and casts and results were plotted as an overall damage score (Fig. 10*C*). Consistent with previous data (Fig. 9), wild-type and *TEC-grem1-cKO* mice displayed similar degrees of damage at *day 2* post-FA exposure (Fig. 10*C*). Interestingly, wild-type, but not *TEC-grem1-cKO*, mice displayed significantly higher kidney damage at *day 14* vs. *day 2*, suggesting that reduced tubular epithelial Grem1 expression attenuates the fibrotic response to FA injury.

**Fig. 11.**
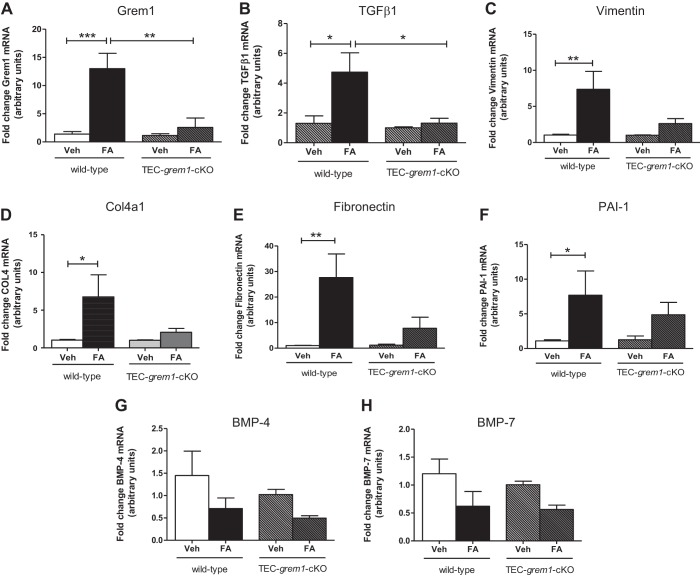
*TEC-grem1-cKO* mice are protected from increases in fibrotic gene expression in the recovery phase of FA-induced nephropathy. RNA was extracted from kidney pole tissues from wild-type and *TEC-grem1-cKO* control and FA-treated mice, cDNA was generated, and quantitative PCR was performed using specific TaqMan probes for Grem1, TGFβ1, vimentin, collagenase type IV α_1_-subunit (Col4a1), fibronectin, plasminogen activator 1 (PAI-1), BMP-4, and BMP-7. Relative quantification was obtained using the ΔΔC_t_ method; 18S and β-actin were used as housekeeping controls. Values (means ± SE) are plotted as fold change for wild-type control (*n* = 7), wild-type FA (*n* = 5), *TEC-grem1-cKO* control (*n* = 4), and *TEC-grem1-cKO* FA (*n* = 4). Control mice were used as the calibrator and set to 1 for each experiment. *P < 0.05, **P < 0.01, ***P < 0.001 (by 1-way ANOVA with Bonferroni's multiple comparison test).

When RNA from kidney poles was analyzed, Grem1 levels were significantly increased in wild-type mice, consistent with previous data showing that Grem1 is a fibrosis-associated gene (28, 33). This increase was abolished in *TEC-grem1-cKO* mice, consistent with the ablation of *grem1* alleles in the tubular epithelial cells (Fig. 11*A*). TGFβ1 expression was significantly increased at *day 14* in wild-type FA-treated, but not in *TEC-grem1-cKO*, mice (Fig. 11*B*). Consistent with an attenuated fibrosis in *TEC-grem1-cKO* kidneys, the extracellular matrix genes Col4a1, fibronectin, and PAI-1 were significantly increased in wild-type FA-treated, but not *TEC-grem1-cKO*, mice (Fig. 11, *D–F*). At *day 14*, vimentin levels were also higher in wild-type kidney only (Fig. 11*C*). BMP-4 and BMP-7 expression levels were not significantly altered in either genotype in response to FA at *day 14* (Fig. 11, *G* and *H*). These data suggest that the milder pathology of the *TEC-grem1-cKO* kidney may be due to attenuated profibrotic gene expression caused by reduced Grem1 and TGFβ1 signaling.

**Fig. 12.**
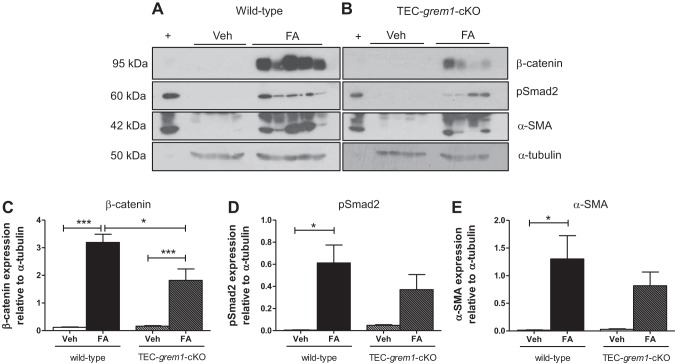
*TEC-grem1-cKO* mice are protected from increases in fibrosis-associated protein expression in the recovery phase of FA-induced nephropathy. *A* and *B*: protein was extracted from kidney medulla from wild-type and *TEC-grem1-cKO* vehicle- and FA-treated mice at *day 14*. Protein lysates (50 μg) were separated by 7.5% or 10% SDS-PAGE and probed via Western blotting using antibodies reactive to β-catenin, phosphorylated Smad2 (pSmad2), pSmad1/5/9, α-smooth muscle actin (SMA), E-cadherin, or α-tubulin. +, Positive control lysate. *C–E*: densitometry analysis with ImageJ software was performed on scanned X-ray films, and band intensities were calculated and plotted as a ratio of antibody intensity normalized to α-tubulin. Values are means ± SE. *P < 0.05, ***P < 0.001 (by 1-way ANOVA with Bonferroni's multiple comparison test).

Consistently, Western blot and densitometry analysis showed significantly increased pSmad2 and α-smooth muscle actin in wild-type FA-treated, but not *TEC-grem1-cKO*, mice at *day 14* (Fig. 12, *A, B, D*, and *E*). β-Catenin levels were significantly increased in both genotypes, but to a much greater extent in wild-type kidney (Fig. 12, *A–C*). These data complement the gene expression data and, again, suggest that the milder pathology of the *TEC-grem1-cKO* kidney may be due to reduced Grem1 and TGFβ1 signaling.

**Fig. 13.**
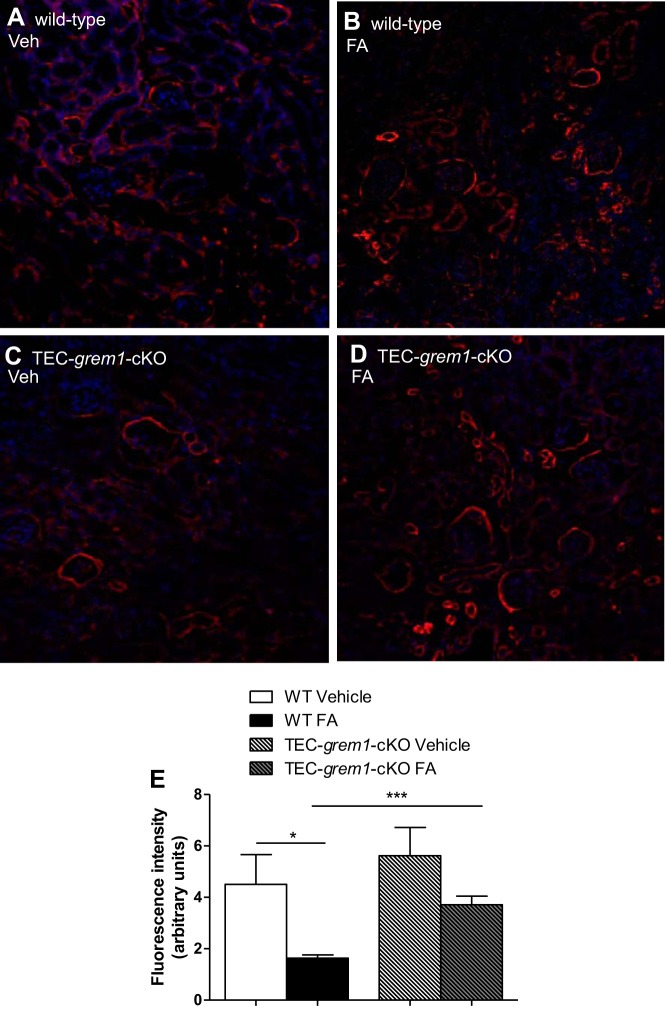
*TEC-grem1-cKO* mice are protected from damage to the renal vasculature. *A–D*: paraformaldehyde-fixed kidney samples were processed and embedded in paraffin wax. Sections were cut at 4 µm and stained for Col4a1 (red) and 4′,6-diaminido-2-phenylindole to stain the nuclei (blue). Stained slides were imaged on a Nikon confocal microscope. Representative images from each group are shown at ×20 magnification. *E*: intensity of the red channel in a defined area of each image (×20 magnification, 3 images per section, *n* = 3 per group). Background intensity was subtracted. Values are means ± SE. *P < 0.05, ***P < 0.001 (by Student’s unpaired *t*-test).

We stained capillaries with Col4a1 to investigate the renal vasculature in wild-type and *TEC-grem1-cKO* mice at *day 14* compared with vehicle control mice (Fig. 13). Col4a1 staining detected capillaries surrounding and within the glomeruli, as well as throughout the kidney (Fig. 13, *A–D*). Wild-type FA-treated mice exhibited significantly reduced fluorescence intensity, indicating a reduction in the number of blood vessels as a result of FA-induced AKI (Fig. 13, *A, B*, and *E*). Interestingly, *TEC-grem1-cKO* mice did not display significant changes in fluorescence intensity and renal vasculature compared with TEC-*grem1*-*cKO* vehicle control mice (Fig. 13, *C–E*). The data suggest that *TEC-grem1-cKO* mice are protected from renal vascular loss associated with FA-induced AKI.

## DISCUSSION

Our data suggest that Grem1 contributes to kidney development and the pathogenesis of AKI. Grem1 levels are low in mouse kidney and highest in the colon (Fig. 1*A*). In contrast, Grem2 levels are highest in mouse brain, suggesting tissue-specific expression patterns for these related proteins (Fig. 1*B*). In human cells, Grem1 levels are high in fibroblasts, MSCs, and macrophages/monocytes (Fig. 2*A*). Grem1 was shown to inhibit BMP2-mediated differentiation of MSCs into osteoblasts (26). Grem1 binding to Slit proteins has been demonstrated to inhibit monocyte chemotaxis and macrophage formation (3, 22, 23). Thus, high levels of Grem1 expression in these cells may represent a normal homeostatic mechanism regulating MSC and monocyte function.

The vast majority of *grem1*^−/−^ mice on a C57BL/6 background die shortly after birth due to renal agenesis and lung defects, emphasizing the critical role in development (1, 10, 19, 20). Using a single backcross onto the FVB genetic background, we were able to generate *grem1*^−/−^ mice that survived to adulthood (~8% of offspring from *grem1*^+/−^ crosses; Table 1). These *grem1^−/−^* mice were smaller than wild-type controls (Fig. 1, *E–G*) and also displayed the previously described fore- and hindlimb defects (Fig. 1, *C* and *D*). The pattern of Grem1 RNA staining in the muscularis layer of the colon is consistent with previous reports (Fig. 3) (7). The low levels of Grem1 detected in kidney epithelium are also consistent with our PCR data (Figs. 1*A* and 6). The ability of a small number of *grem1*^−/−^ mice to survive when the majority die is related to the development of a single, enlarged kidney in the majority of these mice (Fig. 4, Table 2). This larger kidney displayed normal histology with a reduced glomerular density but an overall glomerular number similar to wild-type mice (Fig. 5). Why would the majority of mice develop a single, left kidney in the absence of Grem1? Mammalian kidney development is highly complex and involves formation of the metanephros at embryonic *day 10.5*. Glial-derived neurotrophic factor then triggers the outgrowth of the ureteric bud from the Wolffian duct into a population of nephrogenic cells called the metanephric mesenchyme (6). A range of key genes, such as WT1, Pax2, Pax8, Foxd1, and Six2, along with signaling molecules, such as Wnt and Ret, are involved in kidney development (6). The development of the left kidney in the majority of *grem1*^−/−^ mice may be related to the left-right asymmetry that dominates during embryogenesis. Given the previous dogma that Grem1 was absolutely required for kidney development, the fact that some mice survive with a single kidney suggests that alternate signaling molecules may be able to compensate for the lack of Grem1. The backcross of C57BL/6 mice onto the FVB background may increase the influence of modifier genes that facilitate the development of a single kidney in the absence of Grem1. The increase in Grem2 levels in the *grem1^−/−^* kidney (data not shown) suggests that Grem2 could play a role in compensating for the loss of Grem1 during kidney development. Further work is needed to elucidate why the left kidney develops in the majority of these mice.

Grem1 is reported to drive angiogenesis via activation of VEGFR2 in endothelial cells (4, 21, 27). Others have suggested that Grem1 can activate VEGFR2 in renal epithelial cells and that this pathway contributes to Grem1-induced renal fibrosis (16). No obvious defects in retinal vascularization or visual responses were detected in the *grem1*^−/−^ retina (Fig. 7, and data not shown), suggesting that Grem1 may not play a physiological role in retinal blood vessel formation. No other defects in vascularization of the kidney or other tissues were detected in *grem1*^−/−^ mice (data not shown). In our hands, Grem1-mediated activation of VEGFR2 is very weak compared with VEGF in endothelial colony-forming cells and even weaker in HK-2 kidney epithelial cells (R. Church, unpublished observations). Given the existing data in the literature, it may be that Grem1-mediated activation of VEGFR2 and associated angiogenesis may only occur in pathological conditions involving supraphysiological levels of Grem1.

The successful generation of *TEC-grem1-cKO* mice allowed us to circumvent the challenges of the low numbers of whole body *grem1*^−/−^ mice with a single kidney in our renal injury experiments. The ksp-cadherin-Cre mouse has been used previously to facilitate renal epithelial deletion of genes, including the insulin receptor (32). Successful development of kidneys in these mice is likely due to the appropriate Grem1 expression during ureteric budding, facilitating normal metanephros development and kidney formation. Grem1 levels were significantly lower in kidney poles from *TEC-grem1-cKO* than wild-type mice (Fig. 8*D*), with the residual Grem1 signal likely due to nonepithelial cells in the tissue used for RNA isolation. The concomitant increase in Grem2 expression is interesting and suggests that regulation of Grem1 and Grem2 may be linked to allow compensatory changes in either protein in specific cellular contexts. Although the overall damage score for *TEC-grem1-cKO* mice in response to FA increased at *day 14* compared with *day 2* (Fig. 10*C*), this was not significant and demonstrates for the first time that specific reduction of Grem1 in tubular epithelial cells attenuates the fibrotic response in AKI. Consistently, FA-induced increases in fibrosis-associated genes, such as vimentin, fibronectin, PAI-1, and Col4a1, were reduced in *TEC-grem1-cKO* mice (Fig. 11). Importantly, *TEC-grem1-cKO* FA-treated mice at *day 14* did not display an increase in TGFβ1 expression compared with wild-type FA-treated mice at *day 14*, which showed a nearly fivefold increase (Fig. 11*B*). Protein level measurements at *day 14* showed attenuated β-catenin, pSmad2, and α-smooth muscle actin expression in FA-treated *TEC-grem1-cKO* compared with FA-treated wild-type mice (Fig. 12). These data suggest that *TEC-grem1-cKO* mice are somewhat protected from FA-induced renal fibrosis through decreased TGFβ1 signaling. Furthermore, analysis of results from Col4a1 staining of the renal capillaries showed that *TEC-grem1-cKO* mice were protected from FA-induced damage to the vasculature, which could be an additional mechanism of protection in response to FA injury (Fig. 13). Previous reports demonstrated that tubular epithelial overexpression of Grem via a kidney androgen-regulated promoter aggravated renal damage in FA injury and DN after 25 wk (9, 17). The exact signaling modalities of Grem1 in the diseased kidney are not clear. Increased Smad1/5/9 phosphorylation as a measure of BMP signaling due to a genetic reduction of Grem1 or antibody-mediated inhibition has been reported by our group and others (5, 28). Other reports suggest that activation of VEGFR2 and NFκB inflammatory signaling plays a role in Grem1-mediated kidney damage (16). Other reports again imply that Grem1-mediated signaling in disease occurs via a BMP- and VEGFR2-independent mechanism (14, 25). It is possible that a range of Grem1 signaling may be involved in mediating its profibrotic effects in kidney and other tissues.

Overall, these data contribute to our understanding of the role of Grem1 in AKI, where tubular deletion of Grem1 may have a protective effect, and complement the recent findings by Droguett et al. (9), who showed that tubular overexpression of Grem1 increases renal damage. In the current literature, there is little evidence for a role of BMPs and their antagonists in AKI. Uterine sensitization-associated gene (USAG)-deficient mice display attenuated renal injury and proximal tubular damage in cisplatin-induced nephrotoxicity (34). Furthermore, USAG-1 was found to display the highest BMP antagonist-to-BMP ratio in two models of AKI; a lower ratio was observed with Grem1 (31). This may also implicate USAG-1 as an important protein in AKI. In addition to the range of studies that support the hypothesis of targeting Grem1 in fibrotic conditions (5, 35), these data further enhance our understanding of the molecular basis of Grem1-mediated renal damage and contribute to the body of evidence for Grem1 involvement in renal fibrosis.

## GRANTS

Work on this project was funded by Diabetes UK and a Biotechnology and Biological Sciences Research Council Collaborative Awards in Science and Engineering Award to R. H. Church. D. P. Brazil is funded by the Wellcome Trust; the Department for Employment and Learning, Northern Ireland; and the Northern Ireland Kidney Research Fund.

## DISCLOSURES

No conflicts of interest, financial or otherwise, are declared by the authors.

## AUTHOR CONTRIBUTIONS

R.H.C., I.A., M.T., D.L., A.K., H.M.K., and R.G. performed the experiments; R.H.C., I.A., M.T., D.L., A.K., H.M.K., R.G., and D.B. analyzed the data; R.H.C., I.A., M.T., D.L., A.K., H.M.K., R.G., and D.B. interpreted the results of the experiments; R.H.C., I.A., M.T., D.L., A.K., and D.B. prepared the figures; R.H.C. and D.B. drafted the manuscript; R.H.C. and D.B. edited and revised the manuscript; F.M. and D.B. conceived and designed the research; D.B. approved the final version of the manuscript.
